# Characteristics of skin microbiome associated with disease severity in systemic sclerosis

**DOI:** 10.71150/jm.2409018

**Published:** 2025-01-24

**Authors:** Kyung-Ann Lee, Asad Ul-Haq, Hoonhee Seo, Sujin Jo, Sukyung Kim, Ho-Yeon Song, Hyun-Sook Kim

**Affiliations:** 1 Division of Rheumatobiology, Department of Internal Medicine, Soonchunhyang University Seoul Hospital, Seoul 04401, Republic of Korea; 2 Human Microbiome Medical Research Center (HMMRC), Soonchunhyang University, 22, Soonchunhyang-ro, Sinchang-myeon, Asan, Chungnam 31538, Republic of Korea; 3 Department of Microbiology and Immunology, School of Medicine, Soonchunhyang University, Cheonan-si, Chungnam 31151, Republic of Korea

**Keywords:** systemic sclerosis, microbiome, skin, modified rodnan skin score, diffuse cutaneous

## Abstract

Systemic sclerosis (SSc) is a chronic autoimmune disorder characterised by skin fibrosis and internal organ involvement. Disruptions in the microbial communities on the skin may contribute to the onset of autoimmune diseases that affect the skin. However, current research on the skin microbiome in SSc is lacking. This study aimed to investigate skin microbiome associated with disease severity in SSc. Skin swabs were collected from the upper limbs of 46 healthy controls (HCs) and 36 patients with SSc. Metagenomic analysis based on the 16S rRNA gene was conducted and stratified by cutaneous subtype and modified Rodnan skin score (mRSS) severity. Significant differences in skin bacterial communities were observed between the HCs and patients with SSc, with further significant variations based on subtype and mRSS severity. The identified biomarkers were *Bacteroides* and *Faecalibacterium* for patients with diffuse cutaneous SSc with high mRSS (≥ 10) and *Mycobacterium* and *Parabacteroides* for those with low mRSS (< 10). *Gardnerella*, *Abies*, *Lactobacillus*, and *Roseburia* were the biomarkers in patients with limited cutaneous SSc (lcSS) and high mRSS, whereas *Coprococcus* predominated in patients with lcSS and low mRSS. Cutaneous subtype analysis identified *Pediococcus* as a biomarker in the HCs, whereas mRSS analysis revealed the presence of *Pseudomonas* in conjunction with *Pediococcus*. In conclusion, patients with SSc exhibit distinct skin microbiota compared with healthy controls. Bacterial composition varies by systemic sclerosis cutaneous subtype and skin thickness.

## Introduction

Systemic sclerosis (SSc) is a rare autoimmune connective tissue disorder associated with significant morbidity and mortality, primarily attributable to fibrosis and vasculopathy. SSc affects various organ systems, such as the skin, lungs, gastrointestinal tract, and blood vessels (Denton & Khanna, 2017). Recent studies have elucidated the molecular and immunological alterations distinctive to individuals with SSc (Hinchcliff et al., 2013; Johnson et al., 2015; Mahoney et al., 2015; Milano et al., 2008; Pendergrass et al., 2012). Skin fibrosis is a hallmark of SSc and can be classified as diffuse cutaneous SSc (dcSSc) or limited cutaneous SSc (lcSSc) based on the extent of skin sclerosis. lcSSc is defined as skin thickening distal to the elbows and knees, with or without facial involvement and dcSSc is characterized by skin thickening both distal and proximal to elbows and knees, and may also involve the face (Volkmann et al., 2023). Skin thickness is a surrogate marker for disease activity, severity, and mortality in patients with dcSSc. In early dcSSc, increased skin thickening is typically linked to the onset or worsening of internal organ involvement and elevated mortality risk. Increases in mRSS severity are associated with elevated mortality and adverse renal and cardiac outcomes (Khanna et al., 2017).

Disruptions in the microbial communities on the skin may contribute to the onset of autoimmune diseases that affect the skin (Weyrich et al., 2015). These resident microbes actively interact with other microbes, host epithelium, and immune system, thereby influencing skin health. Bioactive metabolites produced by commensal microbes eliminate pathogens and stimulate keratinocytes and immune cells. This, in turn, modulates both the innate and adaptive immune responses, thereby protecting against toxic and foreign substances. Studies examining the microbiome associated with other inflammatory skin diseases such as psoriasis and atopic dermatitis have demonstrated the potential involvement of skin microbial communities in disease pathogenesis (Boix-Amoros et al., 2023; Koh et al., 2022). However, current research on the skin microbiome in SSc is lacking.

The research conducted thus far has included only a few patients with SSc, and some studies did not include control groups. Investigations of the skin microbiome in SSc (n = 4) have revealed an increase in abundance of *Rhodotorula glutinis* in the forearm skin of individuals with early dcSSc compared to healthy controls (HCs) (Arron et al., 2014). Furthermore, the skin microbiome of patients with SSc exhibits a decreased abundance of lipophilic bacteria, such as *Cutibacterium* and *Malassezia* fungus, and an increased presence of gram-negative bacteria, including *Burkholderia*, *Citrobacter*, and *Vibrio* (Johnson et al., 2019). However, results are conflicting, with some reports indicating no differences in the skin microbiome based on the severity of skin fibrosis in patients with SSc (Johnson et al., 2019), whereas others suggesting correlations between cutaneous *Alphaproteobacteria* and the modified Rodnan skin score (mRSS) (Russo et al., 2024).

This cross-sectional study aimed to investigate taxonomic and functional differences of the cutaneous microbiomes between patients with SSc and HCs. Furthermore, we aimed to compare skin microbial composition based on cutaneous subtype and skin thickness in patients with SSc.

## Materials and Methods

### Study population

This study included patients with SSc between June and August 2021. Participants were classified according to the 2013 American College of Rheumatology/European League against Rheumatism classification criteria (van den Hoogen et al., 2013). The HCs were age- and sex-matched individuals without history of SSc. Participants meeting the following criteria were excluded: age of < 19 or > 70 years, use of antibiotics or any other probiotic bacterial supplement 3 months prior to the study period; underlying skin diseases (i.e., atopic dermatitis or psoriasis); application of topical steroids or retinoid ointment 2 weeks prior to the study period; overlap with connective tissue diseases; pregnancy; and malignancy. The study was approved by the Institutional Review Board for Human Research (2020-10-021) of Soonchunhyang University Seoul Hospital. Written informed consent was obtained from all participants.

### Clinical and laboratory evaluation

Demographic, clinical, laboratory, and instrumental data were collected for each patient. Patients with SSc were classified as having either dcSSc or lcSSc. The mRSS is a physician-performed assessment measure used to examine extent and progression of cutaneous fibrosis in patients with SSc It has been shown to have acceptable reliability and interobserver variability. Skin involvement was assessed using the mRSS (range: 0–51 over 17 body sites), which uses a scale of 0–3 (0, normal; 1, mild thickening; 2, moderate thickening; and 3, severe thickening) on the date of skin swabbing (Khanna et al., 2017).

We investigated digital ulcers and internal organ involvement in patients with SSc, including interstitial lung disease (ILD) and gastrointestinal involvement, in patients with SSc. Laboratory findings, including the presence of anti-topoisomerase, anti-centromere, anti-RNA polymerase III, anti-Ro/SSA, and anti-RNP antibodies, were also recorded.

### Sample collection

Skin swabs were obtained from the dorsal aspect of the participant’s left forearm. The participants were instructed to avoid washing or using any cosmetics on the area for 12 h before sample collection. Samples were collected using an NBgene-SKIN (NBG-S22S) skin sampling kit (Noble Biosciences Inc., Korea). The participants were swabbed 20 times using fresh sterile gloves. After collection, the samples were immediately labelled and stored at -80°C. They were transported on dry ice to the Probiotic Microbiome Convergence Center of Soonchunhyang University (Korea).

### DNA extraction, polymerase chain reaction (PCR), and sequencing

DNA was extracted using a QIAamp DNA Quick Stool Mini Kit (Qiagen, Germany) following the manufacturer's instructions. DNA concentrations were determined using a Qubit-4 fluorometer (Thermo Fisher Scientific, UK), and DNA quality was assessed using 0.8% agarose gel electrophoresis. All DNA samples were stored at -20°C for further examination.

The detailed method for 16S rRNA amplicon PCR has been previously described by our team (Ul-Haq et al., 2022a, 2022b). Briefly, PCR amplification was performed using the described Illumina 16S rRNA amplicon primer set (Forward primer: 5´-TCGTCGGCAGCGTC-AGATGTGTATAAGAGACAGCCTACGGGNGGCWGCAG-3´, Reverse primer: 5´-GTC-TCGTGGGCTCGGAGATGTGTATAAGAGACAGGACTACHVGGGTATCTAATCC-3´) targeting the V4 region to amplify a single span of approximately ~460 bp, with each primer at a concentration of 5 µmol. Template DNA (10 ng) was mixed with KAPA HiFi HotStart ReadyMix (Kapa Biosystems). All samples were subjected to PCR, which was performed using an Applied Biosystems Veriti 96-well thermal cycler. Positive controls consisted of 5 µg of human faeces DNA, whereas negative controls lacked the template DNA. A preliminary denaturation stage was conducted at 95°C for 3 min, 25 cycles of denaturation at 95°C for 30 sec, annealing at 55°C for 30 sec, and extension at 72°C for 30 sec, culminating in a final extension step at 72°C for 5 min. The PCR products were purified using Beckman Coulter AMPure beads according to the manufacturer's instructions. Indexing was performed using 5 µl of the purified amplicon PCR result from each sample. The Nextera XT DNA Library Prep Kit (Illumina) and AMPure beads were used for index PCR and PCR cleanup, respectively. The DNA concentration of each sample was adjusted to 1 nM using H_2_O, and 5 μl of each sample was combined. The resulting pooled library was sequenced on an Illumina iSeq100 platform with a 30% PhiX spike. The sequences have been deposited in the Sequence Read Archive (https://www.ncbi.nlm.nih.gov/sra/) and accessed using the Bioproject. Illumina sequences were deposited in the Sequence Read Archive (SRA) (BioProject ID: PRJNA1189347). These sequences are available at http://www.ncbi.nlm.nih.gov/bioproject/1189347.

### 16S rRNA gene-based microbiome analysis

The iSeq100 Illumina FASTQ reads were examined using QIIME 2 2021.11 (Bolyen et al., 2019). The q2-demux plugin was used to detect, correct, and filter sequences based on quality. DADA2 was used to denoise data (Callahan et al., 2016). Any features that were present fewer than five times were removed using the QIIME 2 command line. Mafft was utilised to align unique amplicon sequence variants (ASVs), and fasttree2 was used to create a phylogenetic tree based on q2 phylogenies (Katoh et al., 2002; Price et al., 2010). The q2-diversity command line was used to calculate alpha diversity measures, including the Faith’s phylogenetic diversity, observed features, Shannon diversity, and beta diversity (Bray-Curtis and Jaccard matrices), following rarefaction of samples to 4000 reads using the default QIIME2 parameters. At different taxonomic levels, ASVs were aligned with the Greengenes reference database (version 13-8) (Arndt et al., 2012) to determine their classification. The taxonomic composition of each individual was analyzed using the METAGENAssist online server (Arndt et al., 2012).

We used the linear discriminant analysis effect size (LEfSe), a statistical method proposed by Segata et al. (2011), to identify discriminatory data between groups. In addition, we performed microbiome clustering, correlation, and network analyses using MicrobiomeAnalyst, a web-based platform described by Chong et al. (2020). To predict functional profiles, we employed the Kyoto Encyclopedia of Genes and Genomes (KEGG) (Ye & Doak, 2009) and Phylogenetic Investigation of Communities by Reconstruction of Unobserved States (PICRUSt2) (Douglas et al., 2020).

### Statistical analysis

Differences in average relative abundance of various taxonomic groups were determined using the Kruskal-Wallis non-parametric test (*p ≤ 0.05, **p ≤ 0.01, and ***p ≤ 0.005) using the GraphPad software, Inc. The mean percentage taxonomic compositions were determined using the STAMP software (Parks et al., 2014) in conjunction with Welch’s t-test. Taxa with the least variation at the significance level of *p = 0.05 were reported. Alpha diversity among groups was evaluated using an ordinary one-way analysis of variance. The significance of beta diversity was determined via permutational multivariate analysis of variance, with differences at a significance level of *p = 0.05. The LEfSe approach was used with a linear discriminant analysis (LDA) score of 2 to assess microbial community differences by leveraging the Galaxy interface (https://huttenhower.sph.harvard.edu/galaxy). Differences in functional profiles among the three groups (dcSSc, lcSSc, and HC; SSc with mRSS ≥10, SSc with mRSS <10, and HC) were evaluated using the STAMP software (Parks et al., 2014) and Welch’s t-test. Statistical significance was set at *p* < 0.05 in all analyses.

## Results

### Baseline characteristics

Altogether, 36 patients with SSc (32 women and 3 men) [mean (standard deviation, SD) disease duration: 3.0 (2.3) years; mean (SD) age 51.2 (12.8) years] and 46 HCs were enrolled. All participants recruited for the study were Korean. Among the patients with SSc, 16 (42.1%) had dsSSc, and 22 (57.9%) had lcSSc. The baseline characteristics of the study population are shown in Table S1. No significant differences in age, sex, or smoking habits were observed between the SSc and control groups. On physical examination, mean (SD) mRSS was 9.2 (8.7) in the enrolled SSc patients: 13.8 (11.0) in the dcSSc subgroup and 5.5 (3.6) in the lcSSc subgroup (p=0.0144). Patients with dcSSc had more ILD and positive anti-Scl70 and RNA polymerase III results. Patients with lcSSc had more secondary SS and positive anti-centromere results. Regarding current therapies, only 27.2% (6/22) of the patients with lcSSc received immunosuppressants, compared to 75% (12/16) of the patients with dcSSc. The daily dose of glucocorticoids was higher in patients with dcSSc than in those with lcSSc [5.4 (2.9) mg/day vs. 1.3 (2.0) mg/day].

### Sequencing results

DNA sequencing of all 82 skin samples (36 patients with SSc and 46 HCs) produced between 4,314 and 112,711 reads, with median frequencies of 38,362 to 3,337,503 high-quality clean paired-ends.

### Comparison between HCs and patients with SSc

**Fig. 1.**
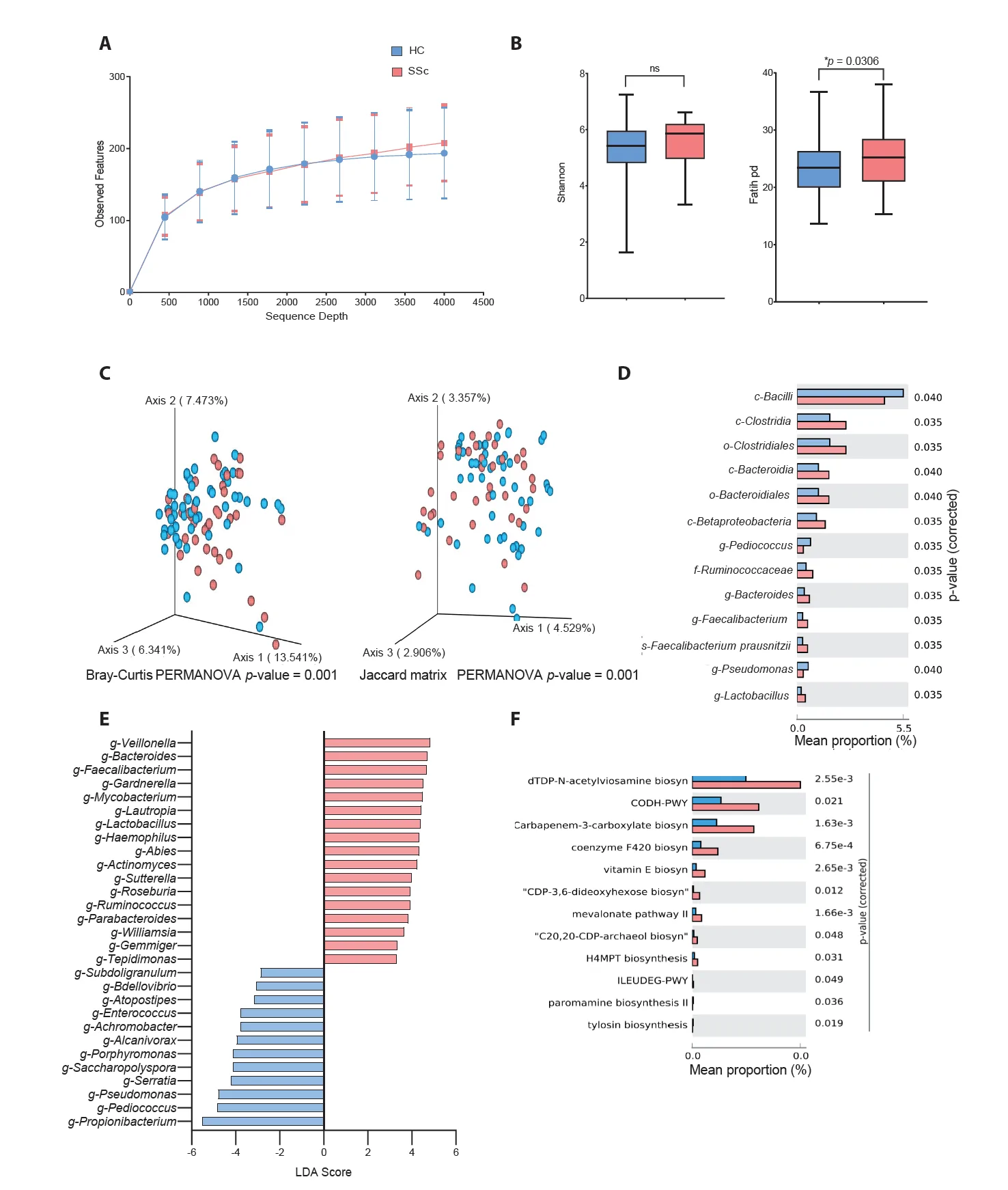
Comparison between healthy controls (HCs) and patients with systemic sclerosis (SSc). (A) Sequencing sample depth and observed features. Taxa sequences were not significantly different between the two groups. (B) The alpha diversity includes Shannon diversity (left panel) and Faith phylogenetic diversity (right panel). (C) Beta diversity analysis based on the Bray–Curtis and Jaccard matrices showed significant differences between the HC and SSc groups (*p < 0.05). (D) Mean percentage population differences between the HC and SSc groups at different taxa levels. Statistical significance between groups was analysed by Welch’s t-test using the STAMP software (*p < 0.05). (E) The taxonomic figure was derived from a linear discriminant analysis effect size analysis with linear discriminant analysis scores of > 2 and significance at *p < 0.05 as determined using the Kruskal-Wallis test. (F) Mean proportion for KEGG pathways abundance between the HC and SSc groups. Statistical significance between groups was analysed by Welch’s t-test using the STAMP software at a threshold level of *p = 0.05 and effect size filter of 2.0.

Figure 1 shows the differences between HC and SSc groups. The alpha refraction curves show no differences between groups (Fig. 1A). Similarly, alpha diversity analysis (Fig. 1B) indicated no significant differences between the groups, except for the Faith phylogenetic diversity analysis (*p* = 0.030). By contrast, beta diversity analysis based on Bray-Curtis (*p* = 0.001) and Jaccard matrices (*p* = 0.001) indicated significant differences between the groups (Fig. 1C).

Fig. S1 shows the percentage of the average taxonomic composition of HCs and patients with SSc. Bacterial taxa with relative proportion of > 1% are presented. The five most abundant phyla in both groups were Actinobacteria, Firmicutes, Proteobacteria, Firmicutes, Bacteroidetes, and Fusobacteria (Table S2 & Fig. S1). Differences in mean taxa proportions indicated that four classes (Bacilli, Bacteroida, Betaproteobacteria, and Clostridia), two orders (Bacteroides and Clostridiales), one family (*Ruminococcaceae*), five genera (*Bacteroides*, *Faecalibacterium*, *Lactobacillus*, *Pediococcus*, and *Pseudomonas*), and one species (*Faecalibacterium prausnitzii*) differed between HCs and patients with SSc (Fig. 1D). c-Bacilli, g-*Pediococcus*, and g-*Pseudomonas* were common in the HCs, whereas, c-Bacteroida, c-Betaproteobacteria, c-Clostridia, o-Bacteroidal, o-Clostridiales, f-*Ruminococcaceae*, g-*Bacteroides*, g-*Faecalibacterium*, g-*Lactobacillus*, and s-*Faecalibacterium prausnitzii* were abundant in the patients with SSc.

In terms of average percentage composition, c-Bacilli was the most prominent taxon in the HCs (Fig. 1D). Meanwhile, c-Clostridia and o-Clostridiales were more abundant in the patients with SSc. In addition, the LEfSe analysis at the genus level showed that g-*Cutibacterium*, g-*Pediococcus*, and g-*Pseudomonas* were prominent in the HCs, whereas g-*Veillonella*, g-*Bacteroides*, and g-*Faecalibacterium* were abundant in the patients with SSc (Fig. 1E). Furthermore, we predicted the KEGG pathway for both groups, and the mean percentage comparison showed that dTDP-N-acetylviosamine biosynthesis, followed by the carbon monoxide dehydrogenase (CODH) pathway and carbapenem3-carboxylate biosynthesis were the three most prominent pathways in the patients with SSc (Fig. 1F).

### Microbial differences in cutaneous subtypes of SSc

**Fig. 2.**
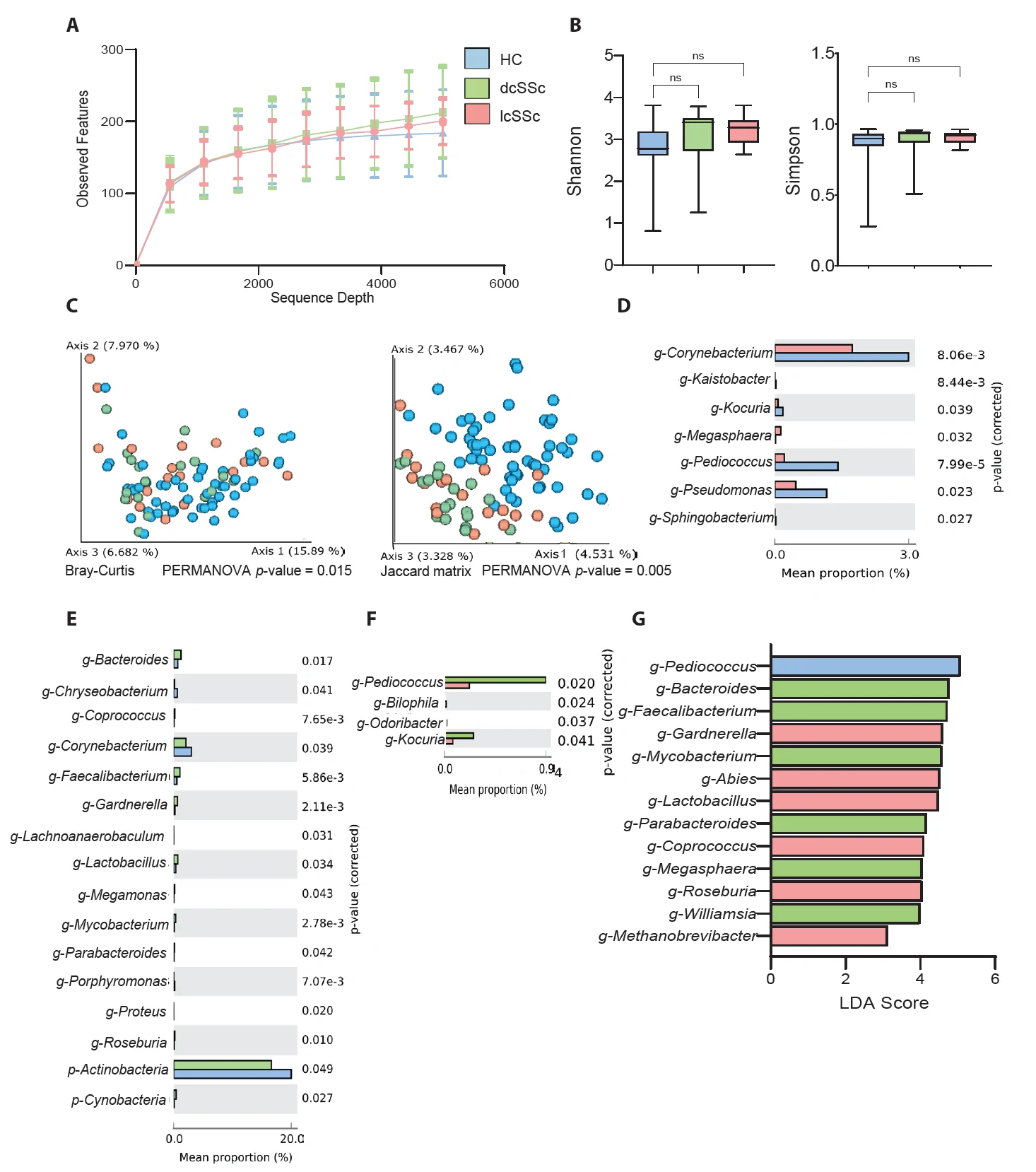
Comparison between healthy controls (HCs) and patients with systemic sclerosis (SSc) according to cutaneous subtypes (lcSSc: limited cutaneous SSc; dcSSc: diffuse cutaneous SSc). (A) Sequencing sample depth and observed features. Groups showed no significant difference in taxa sequences. (B) The alpha diversity includes the Shannon diversity (left panel) and Simpson method (right panel). (C) Beta diversity analysis based on the Bray–Curtis (left panel) and Jaccard matrices (right panel). Significant differences between the HC and the SSc subtypes (lcSSc and dcSSc) (*p < 0.05). (D) Mean percentage population differences between the HCs and dcSSc subgroup. (E) Mean percentage population differences between the HCs and lcSSc subgroup. (F) Mean percentage population differences between the dcSSc and lcSSc subgroups. The groups were compared at genus levels, and significance between the groups was analysed by Welch’s t-test using the STAMP software (*p < 0.05). (G) Comparison between all three groups derived from a linear discriminant analysis effect size analysis with linear discriminant analysis scores of > 2 and significance at *p < 0.05 as determined using the Kruskal-Wallis test.

To compare HCs with patients having different subtypes of SSc (lcSSc and dcSSc), we conducted the same analyses as described above. Sequence sample depth and observed features did not indicate any significant differences among the three groups (Fig. 2A). Alpha diversity analysis using Shannon diversity and Simpson’s methods showed no significant differences among the groups (Fig. 2B). Beta diversity analysis based on the Bray-Curtis and Jaccard matrices showed significant differences among the three groups (*p* < 0.05) (Fig. 2C). The mean percentage of population differences between the HCs and patients with lcSSc showed that g-*Corynebacterium*, g-*Pediococcus*, and g-*Pseudomonas* were prominent in the HCs (Fig. 2D). However, g-*Megasphaera* was abundant in the lcSSc cohort. Similarly, p-Actinobacteria, g-*Corynebacterium*, and g-*Chryseobacterium* were prominent in the HCs (Fig. 2E), whereas, g-*Bacteroides*, g-*Faecalibacterium*, and g-*Gardnerella* were abundant in the dcSSc subgroup. Fig. 2F shows the mean percentage population differences between the dcSSc and lcSSc subgroups. g-*Pediococcus* and g-*Kocuria* were abundant in the dcSSc subgroup, whereas g-*Bilophila* and g-*Odoribacter* were predominant in the lcSSc subgroup. Fig. 2G demonstrates the comparison between the three groups derived from the LEfSe analysis with LDA. g-*Pediococcus* was the most prominent taxon in the HCs. Meanwhile, g-Bacteriodes and g-*Faecalibacterium* had higher LDA scores in the dcSSc subgroup.

### Microbial differences based on the assessment of skin thickness

**Fig. 3.**
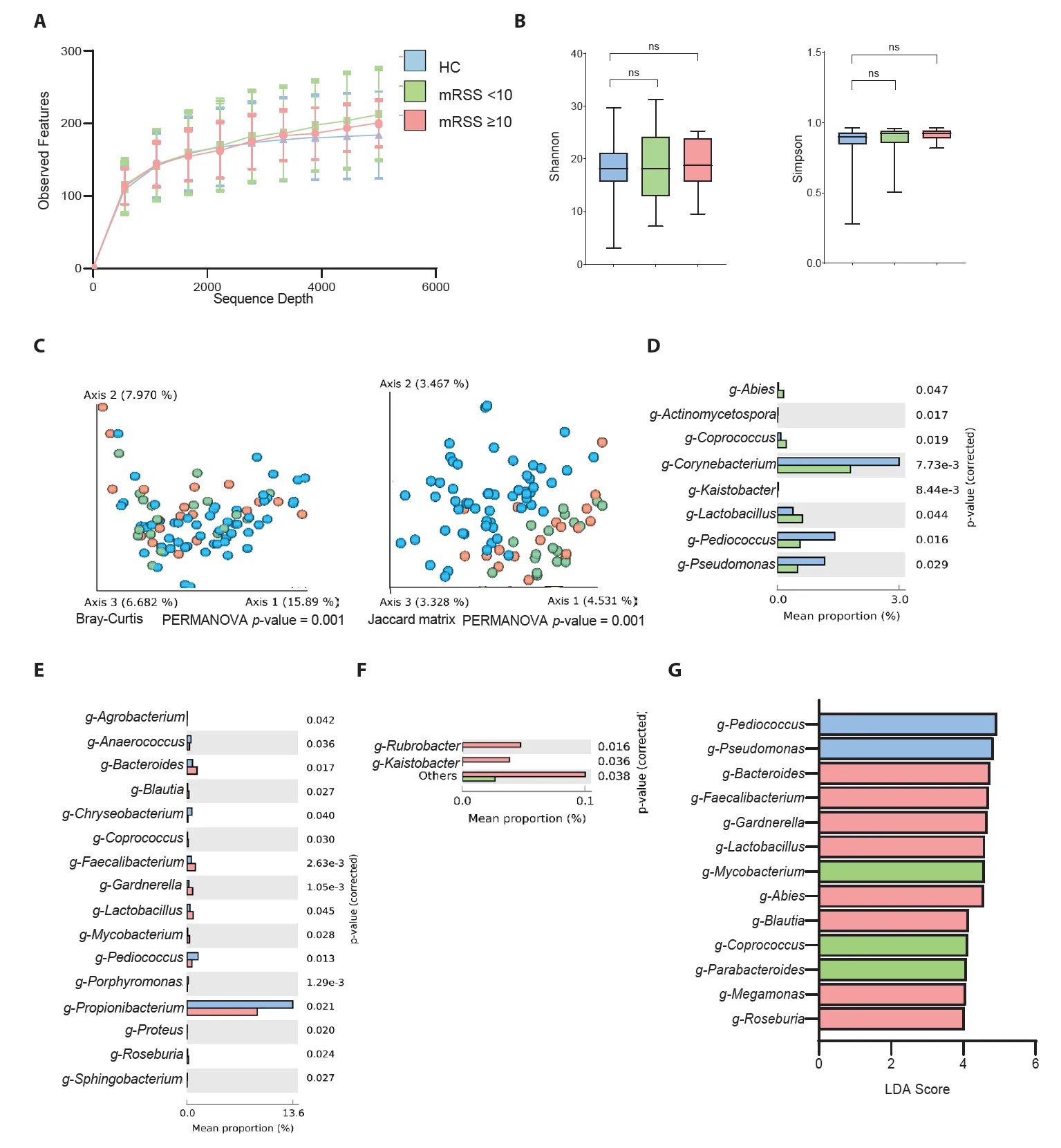
Comparison between healthy controls (HCs) and patients with systemic sclerosis (SSc) according to the modified Rodnan skin score (mRSS < 10 or mRSS ≥ 10). (A) Sequencing sample depth and observed features. Groups showed no significant differences in taxa sequences. (B) The alpha diversity includes Shannon diversity (left panel) and Simpson method (right panel). (C) Beta diversity analysis based on the Bray–Curtis (left panel) and Jaccard matrix (right panel). The data indicate significant differences among the HCs and patients with mRSS < 10 and mRSS ≥ 10 (*p < 0.05). (D) Mean percentage population differences between the HC and mRSS < 10 groups. (E) Mean percentage population differences between the HC and mRSS ≥ 10 groups. (F) Comparison between patients with mRSS < 10 and mRSS ≥ 10. The groups were compared at genus levels, and statistical significance between the groups was analysed by Welch’s t-test using the STAMP software (*p < 0.05). (G) Taxonomic figure derived from a linear discriminant analysis effect size analysis with linear discriminant analysis scores of > 2 and significance at *p < 0.05 as determined using the Kruskal-Wallis test.

The impact of skin hardening on skin microbiome communities was analyzed for its impact on mRSS severity by comparing HC and SSc patients with mRSS values < 10 and ≥ 10 (Fig. 3). This threshold, consistent with previous studies, was found to be a clinically meaningful cutoff, enabling statistically valid analyses and facilitating comparisons with prior research (Khanna et al., 2019). The corresponding distributions are presented in Fig. S2A. Initially, the sequence depth analysis showed no significant differences between the groups (Fig. 3A). Alpha diversity analysis by the Shannon diversity and Simpson methods showed no significant changes among the three groups (Fig. 3B). However, the beta diversity analysis using both Bray–Curtis and Jaccard distributions was distinct among the groups (Fig. 3C). Furthermore, the mean percentage of population differences between HCs and patients with an mRSS value of < 10 indicated that g-*Corynebacterium*, g-*Pediococcus*, and g-*Pseudomonas* were common in the control group (Fig. 3D). Meanwhile, g-*Lactobacillus* was more prevalent in patients with SSc and those with an mRSS value of < 10. However, the comparison between mean percentage population differences between the HCs and patients with an mRSS value of ≥ 10 presented a higher percentage of g-*Cutibacterium* and g-*Pediococcus* in the control group and g-*Bacteroides* and g-*Faecalibacterium* in the patients with SSc with an mRSS value of ≥ 10 (Fig. 3E). Furthermore, Fig. 3F shows a comparison among the SSc groups, indicating the predominance of g-*Rubribacter* and g-*Kaistobacter* in patients with SSc with an mRSS value of ≥ 10 compared with those with an mRSS value of < 10. Moreover, the LEfSe analysis among three groups revealed that g-*Pediococcus* was the most prominent taxon in HCs and that g-*Bacteroides* was prevalent in patients with an mRSS value of ≥ 10 (Fig. 3G). Subsequent analyses of biomarker taxa abundance correlation with disease severity classified by mRSS in SSc patients showed that *Pediococcus*, which had the highest LDA score in the control group, and *Bacteroides*, a biomarker associated with the severe group (mRSS ≥ 10), corresponded with disease severity distribution patterns (Fig. S2B).

## Discussion

**Fig. 4.**
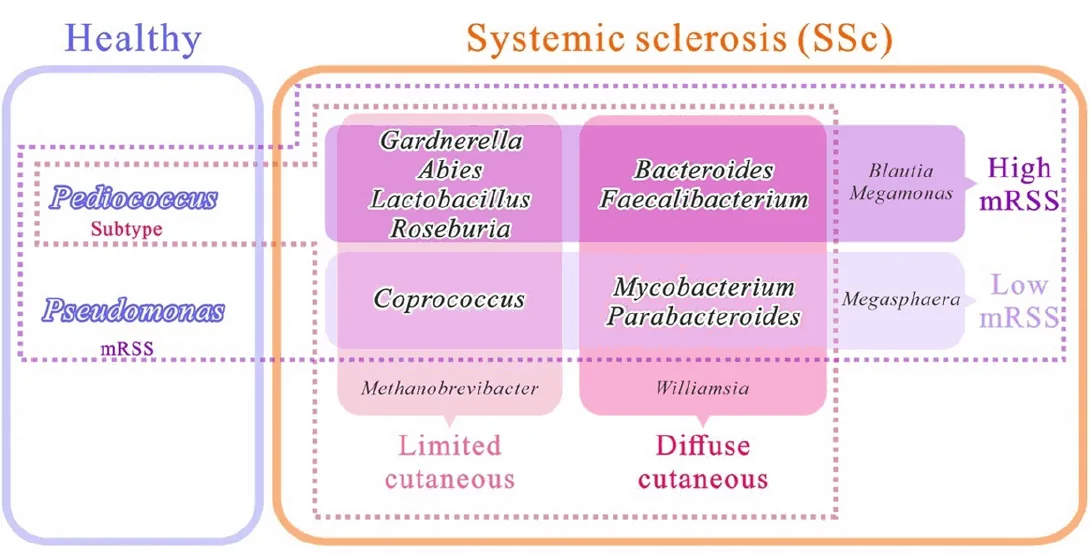
Summary of skin microbiome difference in systemic sclerosis patients compared to healthy controls, based on cutaneous subtype and modified Rodnan skin score (low mRSS < 10 or high mRSS ≥ 10).

SSc commonly affects the skin, and approximately 95% of patients with SSc experience skin fibrosis (Bellocchi et al., 2022; Jaeger et al., 2016). Although a small number of patients present with scleroderma sine scleroderma, a rare form of the disease characterized by internal organ involvement without skin thickening, most patients develop skin fibrosis during their disease (Diab et al., 2014). Several studies have elucidated the alteration of skin microbial composition in patients with SSc. However, a detailed study encompassing different aspects of patients with SSc is lacking. In the present study, we performed 16S rRNA amplicon sequencing of skin samples from a diverse cohort of patients with SSc and HCs. We then compared the microbial composition and function between the control group and patients with SSc, and the control group versus patients with SSc classified according to various subtypes, such as cutaneous subtype and progression of skin thickness (Fig. 4). The characteristics of mRSS in our study (mean mRSS: 13.8 in dcSSc and 5.5 in lcSSc) are consistent with those reported in a previous Korean multicenter cohort study (mean mRSS: 13.2 in dcSSc and 4.3 in lcSSc) (Lee & Moon, 2022; Moon et al., 2018). This study thus presents the general characteristics of Korean patients with SSc.

The populations of g-*Cutibacterium* and g-*Pediococcus* were more abundant in the control group than in the SSc group. A previous study has also showed the patients with SSc exhibited a decreased number of lipophilic taxa, including-g-*Cutibacterium* (Johnson et al., 2019). The predominant microbiome changes according to multiple factors such as local skin anatomy, pH, and local lipid and moisture levels. Sebaceous areas such as the face and back contain high proportions of *Cutibacterium* and *Staphylococcus* species, whereas dry areas such as the forearm contain low levels of *Cutibacterium* (Grice et al., 2009). Our study confirmed that skin changes in patients with SSc contribute to the reduction of g-*Cutibacterium* levels. Interestingly, when comparing patients with SSc and HCs, as well as among SSc subgroups according to cutaneous subtypes and mRSS and HC, g-*Pediococcus* demonstrated a higher proportion in the HC group. Thus, g-*Pediococcus* may be an important genus for the inhibition of SSc. *Pediococcus* is a genus of gram-positive homofermentative lactic acid bacteria (LAB), and some strains have attracted attention for their potential probiotic properties. Certain strains of g-*Pediococcus* strains exhibit antimicrobial activity (Martino et al., 2013), and the antimicrobial effect of LAB is primarily attributed to the production of bacteriocins (Fugaban et al., 2022; Todorov & Dicks, 2009). In addition, some g-*Pediococcus* strains exhibit immunomodulatory properties by stimulating the production of anti-inflammatory cytokines (interleukin-4 and interleukin-13) and suppressing pro-inflammatory cytokines (tumour necrosis factor-alpha) (Jeong et al., 2020). Previous studies have demonstrated the role of-g-*Pediococcus* in preventing and treating skin diseases, such as atopic dermatitis (Jeong et al., 2020). Our previous study has reported that s-*Pediococcus acidilactici* improves the skin barrier function and reduces transepidermal water loss in healthy human volunteers (Park et al., 2024). g-*Pediococcus* also produces exopolysaccharides, which protect the skin from environmental stressors (Jiang et al., 2021). Thus, g-*Pediococcus* may be a potential probiotic for patients with SSc.

Our study also demonstrates an elevated abundance of *pseudomonas* in HC relative to that in patients with SSc. While *Pseudomonas aeruginosa* is widely recognized as an opportunistic pathogen, it is commonly found in the natural microflora of healthy individuals (Grice et al., 2008). *P. aeruginosa* can reside harmlessly on the skin and in the oral cavity, playing a role in maintaining the microbial balance and preventing colonization by more harmful pathogens (Cogen et al., 2008). Studies have shown that it can inhibit the growth of fungi and other pathogens including *Candida albicans* and *Helicobacter pylori* by producing antimicrobial compounds such as pyocyanin and 1-hydroxyphenazine (Kerr, 1994; Kerr et al., 1999; Krausse et al., 2005). The presence of *pseudomonas* in HCs may thus contribute to the prevention of infections by more virulent organisms, which could explain the higher abundance observed in HCs than in patients with SSc in this study.

Patients with SSc showed an increased proportion of Gram-negative anaerobic bacteria (g-*Bacteroides* and g-*Faecalibacterium*), suggesting that these genera include pathogenic bacteria associated with SSc. In particular, patients with progressive subtypes of SSc, including dcSSc and higher mRSS, exhibited an increased abundance of g-Bacteroides and g-*Faecalibacterium*. Therefore, we considered bacterial genera as possible biomarkers of progressive cutaneous fibrosis in SSc. The abundance of *Bacteroides* in skin samples has been reported in various dermatological diseases, including acne vulgaris (Li et al, 2019), hidradenitis suppurativa (Olunoiki et al., 2022), seborrheic dermatitis (Park et al., 2017), and melanoma (Mekadim et al., 2022). A decreased abundance of Bacteroidetes and *Faecalibacterium* and an increased abundance of Firmicutes have been detected in the gut microbiota of patients with SSc (Kim et al., 2022). *Bacteroides* typically have a complex and beneficial relationship with the host in the gut. For instance, *Bacteroides fragilis*, a human commensal bacterium, facilitates the functional maturation of Treg cells, including the production of IL-10 in mice (Round & Mazmanian, 2010). However, these bacteria can lead to severe pathologies such as bacteremia and abscess formation at various body sites if they escape the gut environment. *Bacteroides* species are notable clinical pathogens identified in most anaerobic infections, with an associated mortality rate of > 19% (Wexler, 2007). The pathogenicity of gram-negative bacteria is primarily attributed to the secretion of membrane vesicles, which play a crucial role in bacterial physiology and pathogenesis (Roier et al., 2016). The skin acts as a physical barrier between the body and environment, preventing pathogen colonization. However, gram-negative bacteria can proliferate excessively in the skin by compromising this barrier. *Bacteroides* play an immune-modulating role in the gut; however, outside the gut, they may play a pathogenic role in SSc in environments such as the skin. We also observed the upregulation of the Carbon monoxide dehydrogenase/acetyl-CoA synthase (CODH/ACS) (or reductive acetyl coenzyme A) pathway, which operates in anaerobic environments to fix carbon dioxide by forming acetyl-CoA, which is subsequently fermented into acetate (Diender et al., 2015). A differential metabolic pathway related to the anaerobic bacterial profile also suggests a functional role for Gram-negative taxa in SSc skin. Therefore, further mechanistic studies are required to investigate the effects of gram-negative bacteria, including *Bacteroides* and *Faecalibacterium* on skin fibrosis and inflammation.

Our predictive functional analysis also revealed the enrichment of the N-acetylviosamine biosynthesis pathway in the SSc cutaneous microbiome. Since N-acetylviosamine is a component of the O-antigen, which forms a part of the lipopolysaccharide (LPS) structure (Marolda et al., 1999), our findings align with those of a recent study reporting an increased concentration of LPS in the serum from patients with SSc (Stec et al., 2023). The activation of Toll-like receptor 4 (TLR-4) by LPS triggers the release of key pro-inflammatory cytokines (Lu et al., 2008). The crucial involvement of TLR-4 in pathological tissue fibrosis has been highlighted in murine models of SSc. Furthermore, the elevated expression of interleukin-6 in fibroblasts, endothelial cells, and immune cells in response to LPS in vitro was significantly diminished in the absence of TLR-4 (Takahashi et al., 2015). Our results support the hypothesis that the cutaneous microbiome can affect inflammation and fibrosis in patients with SSc.

This study has some limitations that should be considered. First, the groups were analysed based on the 16S rRNA gene; however, this technique could not identify specific microbial species or strains. Therefore, additional metagenomic and metabolic approaches are required to establish the relationship between specific bacterial species and their functional roles in SSc regulation. Second, as this was a cross-sectional study, a stimulus–response relationship could not be established between SSc and the skin microbiome. Third, to minimize the effects of humidity and temperature, we limited patient recruitment to the summer months of June to August. However, there are uncontrolled factors such as clothing and sun exposure, which represent limitations of our study. Finally, most patients with dsSSc receive treatment. Including newly diagnosed patients (those who have not received systemic treatment) might have produced different results.

In conclusion, this study highlights the characteristic composition and function of the skin microbiota in patients with SSc compared with that in HCs. We also identified the bacterial composition according to the cutaneous subtype and skin thickness.

## Data Availability

The raw Illumina reads supporting the findings of this study have been deposited in the NCBI Sequence Read Archive (SRA) under BioProject ID: PRJNA1189347 and can be accessed at http://www.ncbi.nlm.nih.gov/bioproject/1189347. Additional data are available from the corresponding author upon request.

## Supplementary Information

Table S1. Baseline characteristics of the study population

Table S2. Average percent taxonomy

Fig. S1. Average percent taxonomy graph

Fig. S2. Abundance analysis of biomarker taxa based on disease severity classified by mRSS in SSc patients
