# Supplementary material for: Characteristics of skin microbiome associated with disease severity in systemic sclerosis
Source: J Microbiol. 2025 Jan 24;63(1):e.2409018. doi: 10.71150/jm.2409018 (PMC13577129; doi:10.71150/jm.2409018)
Supplement: Supplementary file 1 [file jm-2409018-Supplementary-Fig-S1.pdf]

Fig. S1. Average percent taxonomy graph.

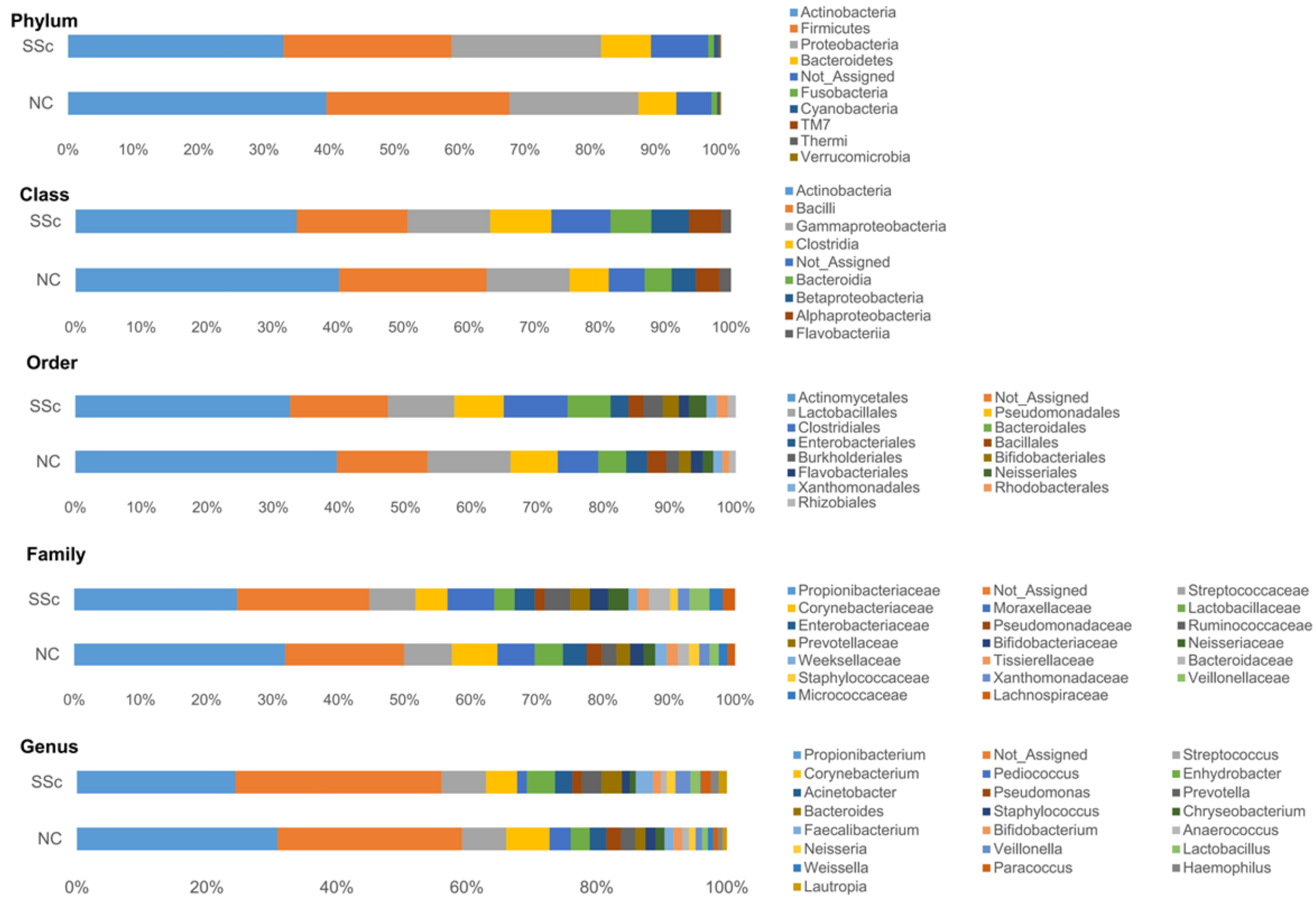

NC Normal control, *SSc* Systemic sclerosis
