# Supplementary material for: Characteristics of skin microbiome associated with disease severity in systemic sclerosis
Source: J Microbiol. 2025 Jan 24;63(1):e.2409018. doi: 10.71150/jm.2409018 (PMC13577129; doi:10.71150/jm.2409018)
Supplement: Supplementary file 2 [file jm-2409018-Supplementary-Fig-S2.pdf]

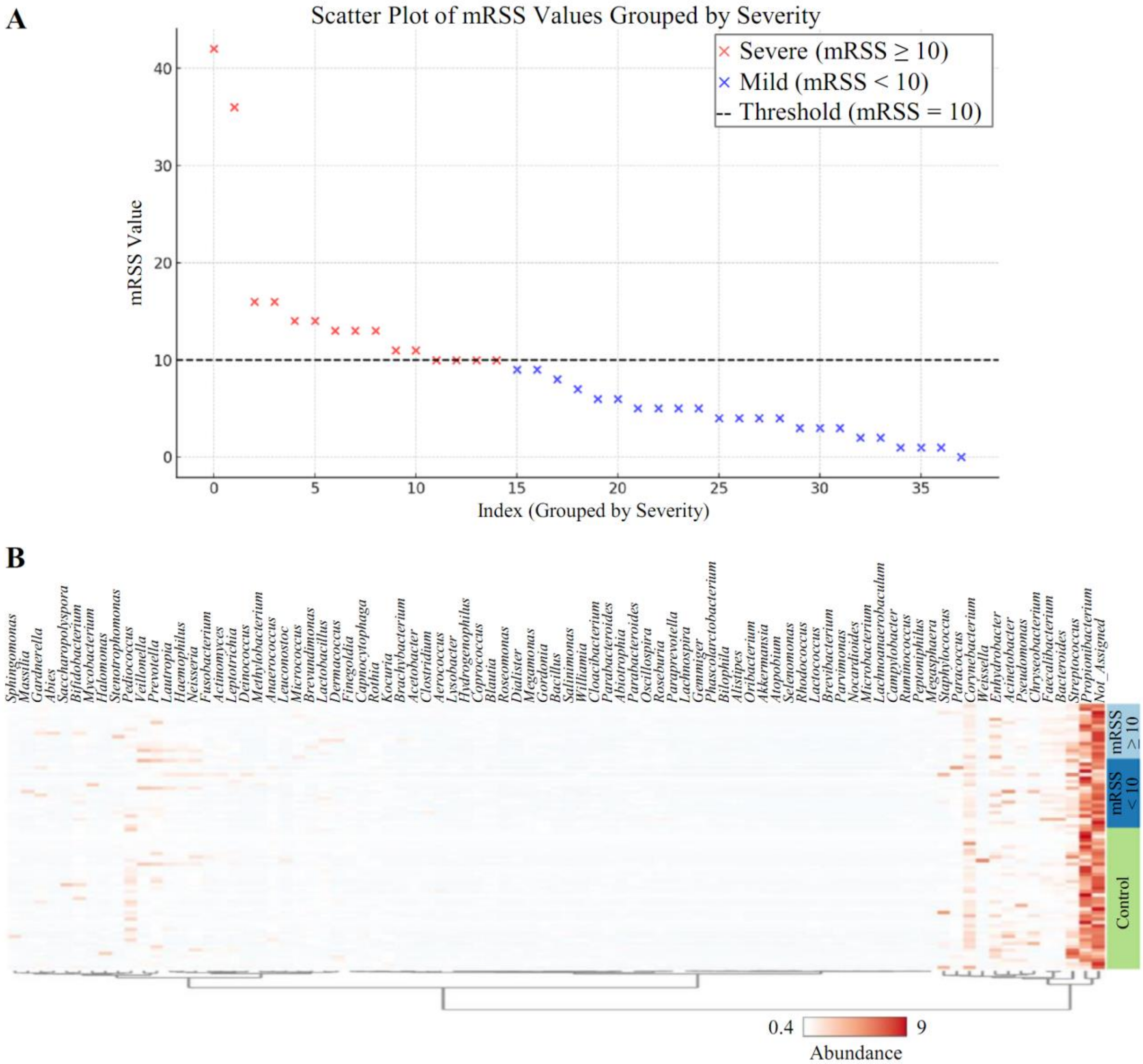

Supplementary Fig. S2. Abundance analysis of biomarker taxa based on disease severity classified by mRSS in SSc patients. (A) In this study, the SSc cohort was divided into two groups based on an mRSS threshold of 10, which was determined to be a clinically meaningful cutoff for evaluating disease severity in patient groups. This decision enabled statistically valid analysis based on data distribution, ensured consistency with the study objectives, and facilitated easier comparison with previous research. (B) Accordingly, the distribution patterns in the normal and patient groups were analyzed. The results highlighted that Pediococcus, which had the highest LDA score in healthy control group, showed greater abundance in the

control group, while Bacteroides, a biomarker associated with the severe group (mRSS $\geq$ 10), corresponded to the disease severity.
