# Supplementary material for: Characteristics of skin microbiome associated with disease severity in systemic sclerosis
Source: J Microbiol. 2025 Jan 24;63(1):e.2409018. doi: 10.71150/jm.2409018 (PMC13577129; doi:10.71150/jm.2409018)
Supplement: Supplementary file 3 [file jm-2409018-Supplementary-Table-S1.pdf]

Table S1. Baseline characteristics of the study population

	Total SSc (n = 38)		dcSSc (n = 16)		lcSSc (n = 22)		Controls (n = 54)	
Age (years)	51.2	(12.9)	51.8	(13.6)	50.8	(12.5)	49.2	(12.4)
Female, n (%)	35	(92.1)	15	(93.7)	20	(90.9)	47	(87.0)
Disease duration, years	3.0	(2.2)	3.0	(2.4)	3.0	(2.2)		
BMI, kg/m^2	21.9	(3.2)	21.5	(3.2)	22.1	(3.1)	23.3	(3.5)
Ever smoker, n (%)	2	(5.3)	1	(6.2)	1	(4.5)	2	(3.7)
Initial mRSS	8.8	(8.5)	13.3	(11.0)	5.5	(3.6)		
Digital ulcer, n (%)	7	(18.42)	5	(31.2)	2	(9.0)		
Interstitial lung disease, n (%)	25	(65.7)	16	(100)	9	(40.9)		
GI involvement, n (%)	21	(55.2)	11	(68.7)	10	(45.5)		
Current medication, n (%)								
Cyclophosphamide	1	(2.6)	1	(6.2)	0	(0)		
Mycophenolate mofetil	17	(44.7)	11	(68.7)	6	(27.2)		
Glucocorticoids (GC)	23	(60.5)	15	(93.7)	8	(36.3)		
Dose of GC, mg/day	3.0	(3.1)	5.4	(2.9)	1.3	(2.0)		
Autoantibodies, n (%)								
Anti-centromere antibody	14	(36.8)	0	(0)	14	(63.6)		
Anti-Scl70 antibody	15	(39.4)	10	(62.5)	5	(22.7)		
Anti-RNA polymerase III	4	(10.5)	4	(36.3)	0	(0)		
Anti-RNP antibody	2	(5.2)	1	(6.25)	1	(4.5)		
Anti-Ro60/SSA	8	(21.0)	3	(18.75)	5	(22.7)		
Anti-Ro52/SSA	9	(23.6)	5	(31.25)	4	(18.1)		

SD standard deviation, *SSc* systemic sclerosis, *dcSSc* diffuse cutaneous SSc, *lcSSc* limiated cutaneous SSc, *mRSS* modified Rodnan total skin score, *GI* gastrointestinal
