# Supplementary material for: Characteristics of skin microbiome associated with disease severity in systemic sclerosis
Source: J Microbiol. 2025 Jan 24;63(1):e.2409018. doi: 10.71150/jm.2409018 (PMC13577129; doi:10.71150/jm.2409018)
Supplement: Supplementary file 4 [file jm-2409018-Supplementary-Table-S2.pdf]

Table S2. Average percent taxonomy

Phylum	HC	SSc
Actinobacteria	39.58	33.01
Firmicutes	28.03	25.74
Proteobacteria	19.76	22.88
Bacteroidetes	5.79	7.63
Not_Assigned	5.38	8.82
Fusobacteria	0.87	0.84
Class		
Actinobacteria	39.54	32.98
Bacilli	22.14	16.57
Gammaproteobacteria	12.47	12.32
Clostridia	5.87	9.13
Not_Assigned	5.38	8.82
Bacteroidia	4.04	6.11
Betaproteobacteria	3.66	5.58
Alphaproteobacteria	3.55	4.88
Flavobacteriia	1.73	1.42
Fusobacteriia	0.87	0.84
Order		
Actinomycetales	37.81	30.67
Not_Assigned	13.14	13.96
Lactobacillales	11.94	9.50
Pseudomonadales	6.83	7.06

Family	HC	SSc
Propionibacteriaceae	27.29	20.41
Not_Assigned	15.41	16.59
Streptococcaceae	6.16	5.81
Corynebacteriaceae	5.90	3.99
Moraxellaceae	4.84	5.86
Lactobacillaceae	3.65	2.56
Enterobacteriaceae	3.03	2.56
Pseudomonadaceae	1.99	1.20
Ruminococcaceae	1.88	3.23
Prevotellaceae	1.78	2.45
Bifidobacteriaceae	1.73	2.31
Neisseriaceae	1.52	2.53
Weeksellaceae	1.50	1.08
Tissierellaceae	1.43	1.50
Bacteroidaceae	1.41	2.63
Staphylococcaceae	1.36	1.01
Xanthomonadaceae	1.31	1.44
Veillonellaceae	1.20	2.49
Micrococcaceae	1.14	1.70
Genus		
Propionibacterium	27.29	20.41
Not_Assigned	25.11	26.56

Clostridiales	5.87	9.13	Streptococcus	6.01	5.73
Bacteroidales	4.04	6.11	Corynebacterium	5.90	3.99
Enterobacteriales	3.03	2.56	Pediococcus	2.86	1.26
Bacillales	2.73	2.16	Enhydrobacter	2.58	3.62
Burkholderiales	1.84	2.75	Acinetobacter	2.25	2.24
Bifidobacteriales	1.73	2.31	Pseudomonas	1.99	1.20
Flavobacteriales	1.73	1.42	Prevotella	1.95	2.55
Neisseriales	1.52	2.53	Bacteroides	1.41	2.63
Xanthomonadales	1.31	1.44	Staphylococcus	1.36	1.01
			Chryseobacterium	1.23	0.75
			Faecalibacterium	1.19	2.24
			Bifidobacterium	1.18	1.01

HC Healthy control, *SSc* Systemic sclerosis
